# Current situation and factors influencing physical fitness among adolescents aged 12 ∼ 15 in Shandong Province, China: A cross-sectional study

**DOI:** 10.1016/j.pmedr.2023.102460

**Published:** 2023-10-12

**Authors:** Zhihao Huang, Shanshan Li, Fei Lu, Kunzong Tian, Lujing Peng

**Affiliations:** a School of Big Data and Fundamental Sciences, Shandong Institute of Petroleum and Chemical Technology, Dongying, China; b Mathematical Group, Chenguan Central Middle School in Guangrao County, Dongying, China; c Physical Education Group, Dongying Experimental Middle School, Dongying, China

**Keywords:** Adolescents, Physical fitness, Qualified rate, Influencing factors

## Abstract

Adolescent physical fitness serves not merely as a current barometer of well-being but as a significant prognosticator of future health trajectories. Amidst the tumult of socioeconomic metamorphoses and pronounced lifestyle transitions enveloping China—mirroring global trends—the imperative to elucidate the present landscape of adolescent physical fitness intensifies. Moreover, discerning the myriad determinants underpinning it becomes paramount. In this context, our research endeavored to meticulously delineate the physical fitness milieu of adolescents residing in Shandong Province, systematically unpacking the multifarious influencers thereof. The insights garnered herein furnish an empirical foundation, primed to guide the sculpting of calibrated interventions, targeting the enhancement of health in this pivotal population cohort. In an extensive evaluative survey conducted in 2023 concerning the physical fitness of Shandong's student populace, a cohort of 33,211 adolescents aged 12 to 15 years was delineated utilizing a stratified random cluster sampling technique. This exercise meticulously quantified the physical fitness indices across diverse gender, age, and household registration classifications, subsequently computing the concomitant qualified rates. Employing multivariable logistic regression analysis, this investigation delved into the determinants modulating the adolescents' physical fitness qualified rate. For 2023, the aggregate fitness qualified rate stood at 91.94 % for the adolescents aged 12 ∼ 15 in Shandong Province. Gender-wise, female adolescents registered a qualified rate of 92.25 %, marginally eclipsing their male peers at 91.63 % (P < 0.05). An age-related trend in qualified rates was discernible, with marked variations across different age bands (P < 0.05): 91.37 % for 12-year-olds, 91.79 % for 13-year-olds, 91.81 % for 14-year-olds, and a zenith of 92.87 % for 15-year-olds. A geographical dichotomy emerged wherein rural adolescents distinctly outperformed their urban counterparts, notching up a 92.28 % qualified rate versus 91.64 % in urban settings (P < 0.05). The multivariable logistic regression analysis showed that after adjusting for gender, age, and household registration characteristics, adolescents had a lower odds of failing the physical fitness tests whose parents both liked physical exercises, whose parents supported children’s participation in physical exercise, who participated in physical exercise sessions 3 ∼ 5 times per week or more than 5 times per week, who exercised for 0.5 ∼ 1 h each time or more than 1 h each time, who engaged in moderate intensity physical exercise, who slept 6 ∼ 8 h per day or more than 8 h per day, who consumed breakfast 3 ∼ 6 times per week or daily. On the other hand, adolescents had a higher odds of failing the physical fitness tests who always exposed to passive smoking, who spent 1 ∼ 3 h on screen per day or more than 3 h on screen per day, who spent more than 3 h doing homework per day, who consumed fast food 2 ∼ 3 times per week or more than 3 times per week. The physical fitness trajectory of adolescents residing within Shandong Province is tethered to a mosaic of determinants. This underscores the imperative for a synergistic strategy, harmonizing parental, scholastic, and societal vectors, to cultivate the salubrious maturation of this pivotal cohort.

## Highlights

• Shandong adolescents aged 12–15 had a 91.94% fitness qualified rate in 2023; females (92.25%) slightly outperformed males (91.63%).
• Age showed a rising fitness qualified trend, peaking at 92.87% for 15-year-olds.
• Rural adolescents surpassed urban peers with 92.28% versus 91.64% fitness qualified rates.
• Positive fitness determinants: parental support for exercise, regular exercise habits, adequate sleep, and frequent breakfast consumption.
• Negative influences: passive smoking exposure, extensive screen time, heavy homework load, and frequent fast food intake.

## 1 Background

In the face of rapid socio-economic changes, there's an increasing emphasis on developing multifaceted talents, particularly those proficient in moral, intellectual, physical, and aesthetic spheres. The physical health of the rising young population has implications beyond personal well-being, influencing their academic performance and playing a role in national progress and overall upliftment. An expanding body of empirical research identifies physical fitness parameters—especially cardiorespiratory capacity and muscular strength—as pivotal factors linked to susceptibility to chronic diseases like cardiovascular diseases, diabetes, and hypertension(Al-Mallah et al., 2018, Elagizi et al., 2018, Henriksson et al., 2020). The risks associated with these are evident even during adolescence (Timpka et al., 2014, Poitras et al., 2016, Henriksson et al., 2019a, Henriksson et al., 2019b). Data suggest that robust cardiorespiratory health during teenage years can offer health benefits persisting into later life (Mintjens et al., 2018, Smith et al., 2014). The American Heart Association, in 2016, highlighted cardiorespiratory endurance as an essential health metric, positioning it alongside traditional indicators such as pulse and blood pressure (Ross et al., 2015). Indeed, a positive correlation exists between physical fitness and overall youth health (Yi et al., 2019). However, growing academic stressors, increased electronic device usage, and decreased physical activity have positioned adolescent health as a significant public health concern (Lissak, 2018, Gao et al., 2022). Globally, the prevalence of overweight and obesity in young populations in 2013 markedly exceeded 1980 figures, showcasing alarming rates in both developed and developing countries (Ng et al., 2014). Although there has been an observed enhancement in the overall physical health of Chinese students over the last decade, specific challenges persist. Notably, there's been a recorded decrease in attributes like flexibility, strength, and power, with only minor advancements in areas such as speed and endurance. Geographical disparities have also emerged, with students in China's eastern regions outperforming their counterparts in the west (Dong et al., 2021). This research intends to scrutinize physical fitness data from 2023, targeting students aged 12 ∼ 15 in Shandong Province. Prioritizing youth health resonates with the United Nations' Sustainable Development Goals for 2030, especially Goal 3, advocating universal health and well-being (Huang and Chang, 2022). This study, while focusing on Shandong's student health, also underscores the broader commitment to achieving these global targets. Recognizing and addressing the determinants of youth health is a crucial stride towards meeting these international objectives, ensuring a healthier future generation ready to champion sustainable progress.

## 2 Materials and methods

### 2.1 Design and participants

**Fig. 1:**
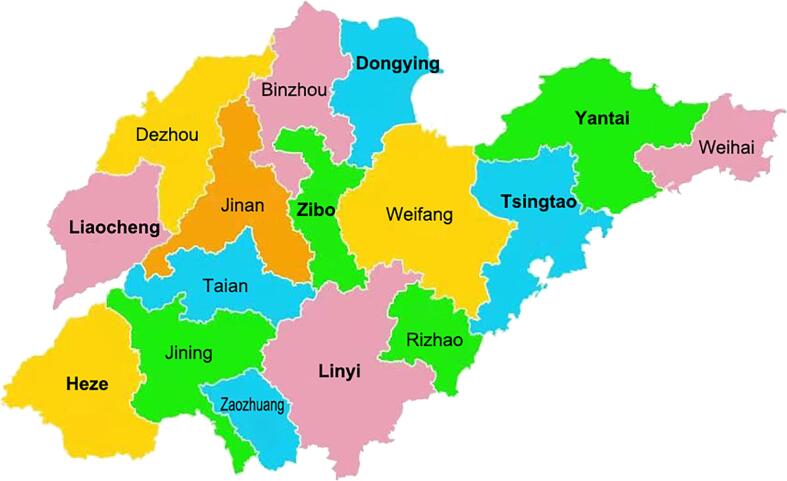
Shandong Province map with surveyed cities highlighted in Black.

In adherence to the recommendations delineated by the Strengthening the Reporting of Observational Studies in Epidemiology guidelines (von Elm et al., 2007), we strategically adopted a stratified random cluster sampling method for our investigation. With an ambition to capture an encompassing profile of Shandong Province, a meticulous selection process was undertaken in May and June of 2023, culminating in the shortlisting of fifty-one middle schools. These schools spanned a diverse array of cities, notably Yantai, Tsingtao, Dongying, Zibo, Linyi, Heze, and Liaocheng. The objective behind this systematic sampling was to facilitate a nuanced representation of the province's student demography (Fig. 1). Concentrating on the student cohort aged 12 ∼ 15 years, the sampling endeavour was bifurcated into two salient stages. Initially, stratification was anchored around the academic grade echelons, thereby safeguarding equitable representation opportunities for each grade. Within the ambit of each selected institution, grades corresponding to our target age bracket were pinpointed, thus demarcating the individual strata. Subsequent to this stratification, we transitioned to the cluster sampling phase. Herein, specific class cohorts within each demarcated grade (stratum) were designated as the sampling clusters. A stochastic selection of classes was then executed from each grade, a move designed to encapsulate students hailing from a gamut of academic and socio-cultural backgrounds. This intricate sampling methodology not only vouched for an unerring representation across grades but also underscored that the selected subsets mirrored the broader scholastic milieu of Shandong. The overarching aim of this approach was to temper biases and curate a sample that, while being diverse, resonated with methodological rigor and precision.

From our survey, which comprised 34,752 students, those presenting significant organ maladies, noticeable physical disabilities or deformities, or those with acute symptoms like colds or fever were excluded. Of the initial cohort, 33,211 students fully participated by undertaking the physical fitness tests and completing the questionnaire, resulting in a participation efficacy of 95.57 %.The gender representation was balanced: boys (n = 16,604) and girls (n = 16,607) each making up 50.00 % of the valid responses. Age stratification revealed 20.80 % (n = 6,909) were 12 years, 24.62 % (n = 8,175) at 13 years, 24.89 % (n = 8,267) at 14 years, and 29.69 % (n = 9,860) were 15 years of age. The overall mean age stood at 13.63 ± 1.114 years.

### 2.2 Measurement of physical fitness and questionnaire survey

Physical fitness tests were meticulously conducted in accordance with the “National student physical health standard (Revised 2014)” (Zhai et al., 2022), sanctioned by the Ministry of Education. The fitness tests were systematically administered in a specific order: firstly, height (centimeter) and weight (kilogram) were measured, followed by the assessment of vital capacity (milliliter). Subsequent tests included the sit-and-reach (centimeter), pull-ups for boys (count) and sit-ups in 1 min for girls (count), standing long jump (centimeter), 50 m sprint (second), and lastly, 1,000 m run for boys (second) /800 m run for girls (second). To determine satisfactory fitness proficiency, an aggregate score of ≥ 60 was established as a benchmark, symbolizing adequacy. This total score is a summation of individual metrics, each given differential weighting. The proportionate emphasis for the fitness parameters is: body mass index (calculated as weight in kilograms divided by the square of height in meters) accounts for 15 %, vital capacity for 15 %, sit-and-reach for 10 %, pull-ups for boys/sit-ups in 1 min for girls for 10 %, standing long jump for 10 %, 50 m sprint for 20 %, and 1,000 m run for boys/800 m run for girls for 20 %. The questionnaire, executed with strict assurances of respondent anonymity, delved into: Demographic characteristics: gender, age, household registration, family annual income (yuan), paternal education level, maternal education level; Home environment: frequency of passive smoking, whether parents liked physical exercise, whether parents supported children's participation in physical exercises; In-school and out-of-school physical exercises: number of physical exercise sessions per week (count), duration of each physical exercise session (hour), intensity of physical exercise; Lifestyle factors: sleep duration per day (hour), screen duration per day (hour), homework duration per day (hour); Dietary behavior: times of breakfast consumption per week, times of meat intake per week, times of vegetable intake per week, times of fruit intake per week, times of eggs intake per week, times of milk intake per week, times of fast food consumption per week (Supplementary Table 1 and Supplementary Table 2).

All testers and investigators in this study were subjected to uniform training, ensuring proficiency in physical fitness tests and field epidemiological investigation methods. Prior to initiating the survey, the students were briefed about the study's objectives and implications. Following the physical fitness tests, questionnaires were immediately dispensed, with students being granted a 30-minute duration for completion. After this interval, questionnaires were expeditiously gathered, methodically organized, and data were subsequently entered for analysis. This study was approved by the Ethics Committee of Shandong Institute of Petroleum and Chemical Technology (registered number: KY-2023–008). All adopted methodologies strictly conformed to the prevailing guidelines and regulations. Additionally, informed consent was duly procured from every participant.

### 2.3 Statistical analysis

Data entry was facilitated using EpiData version 3.1 (EpiData Association), followed by a meticulous data analysis performed with Statistical Product and Service Solutions version 27.0 (International Business Machines Corporation). The continuous variables were delineated as means along with standard deviations, whereas the categorical variables were presented as case counts and percentages (%). The initial phase of analysis involved a univariable analysis, wherein the chi-squared (χ^2^) test served as the primary analytical tool. Variables that demonstrated statistical significance (P < 0.05) during this univariable analysis were subsequently incorporated into a multivariable logistic regression model for a more complex analysis (Bzovsky et al., 2022). This latter stage of analysis employed logistic regression, yielding outcomes quantified through odds ratio (OR), complemented by 95 % confidence interval (CI) to delineate the precision of the estimates. To substantiate the validity of the model, a meticulous assessment of the logistic regression model fit was undertaken, guided by the Hosmer-Lemeshow test, thereby ensuring a robust analytical framework grounded in statistical rigour.

## 3 Results

### 3.1 Physical fitness tests qualified rates among diverse adolescent demographics

From the 2023 survey data concerning adolescents aged 12 ∼ 15 in Shandong Province, the composite physical fitness qualified rate stood at 91.94 %. Crucially, the research delineated a marginally superior qualified rate in females (92.25 %) compared to their male counterparts (91.63 %) (P < 0.05). Additionally, age-specific discrepancies were evident in the qualified rates (P < 0.05): 91.37 % for 12-year-olds, elevating slightly to 91.79 % and 91.81 % for ages 13 and 14, respectively, and peaking at 92.87 % for 15-year-olds. Pertinently, adolescents bearing a rural household registration exhibited a markedly higher qualified rate (92.28 %) compared with those bearing an urban household registration (91.64 %) (P < 0.05) (Table 1).

**Table 1:** Qualified rates according to demographic characteristics of adolescents aged 12 ∼ 15 in Shandong Province who participated in 2023 physical fitness tests and questionnaire survey.

Demographic characteristics	Number of participants who passed the physical fitness test(%)	Number of participants who failed the physical fitness test(%)	χ^2^	*P*
Gender			4.249	0.039
Male	15,215(91.63)	1,389(8.37)		
Female	15,320(92.25)	1,287(7.75)		
Age (year)			8.804	0.032
12	6,313(91.37)	598(8.63)		
13	7,504(91.79)	671(8.21)		
14	7,590(91.81)	677(8.19)		
15	9,128(92.58)	732(7.42)		
Household registration			4.472	0.034
Urban household registration	16,021(91.64)	1,461(8.36)		
Rural household registration	14,514(92.28)	1,215(7.72)		
Family annual income (yuan)			2.538	0.468
≤ 100,000	10,409(91.66)	947(8.34)		
100,001 **∼** 200,000	13,719(92.02)	1,189(7.98)		
200,001 **∼** 300,000	3,977(92.06)	343(7.94)		
≥ 300,000	2,430(92.50)	197(7.50)		
Paternal education level			1.857	0.395
Junior high school or below	5,576(91.51)	517(8.49)		
Senior high school	9,064(92.01)	787(7.99)		
Above senior high school	15,895(92.05)	1,372(7.95)		
Maternal education level			1.744	0.418
Junior high school or below	5,947(91.65)	542(8.35)		
Senior high school	8,314(91.81)	742(8.19)		
Above senior high school	16,274(92.12)	1,392(7.88)

**Table 2:** Univariable analysis of qualified rate among adolescents aged 12 ∼ 15 in Shandong Province who participated in 2023 physical fitness tests and questionnaire survey.

Independent variables	Number of participants who passed the physical fitness test(%)	Number of participants who failed the physical fitness test(%)	χ^2^	*P*
Frequency of passive smoking			11.277	0.004
Never	19,339(92.24)	1,627(7.76)		
Sometimes	7,554(91.80)	675(8.20)		
Always	3,642(90.69)	374(9.31)		
Whether parents liked physical exercise			6.793	0.033
Neither side of the parents liked	10,190(91.46)	951(8.54)		
One side of the parents liked	13,871(92.03)	1,202(7.97)		
Both sides of the parents liked	6,474(92.53)	523(7.47)		
Whether parents supported children's participation in physical exercise			5.923	0.015
Nonsupporting	2,137(90.63)	221(9.37)		
Supporting	28,398(92.04)	2,455(7.96)		
Number of physical exercise sessions per week			47.638	0.000
< 3	6,615(90.02)	733(9.98)		
3 **∼** 5	20,489(92.43)	1,679(7.57)		
> 5	3,431(92.86)	264(7.14)		
Duration of each physical exercise session (hour)			34.218	0.000
< 0.5	6,697(90.32)	718(9.68)		
0.5 **∼** 1	20,616(92.38)	1,700(7.62)		
> 1	3,222(92.59)	258(7.41)		
Intensity of physical exercise			6.277	0.043
Low intensity	2,541(90.72)	260(9.28)		
Moderate intensity	26,400(92.07)	2,275(7.93)		
High intensity	1,594(91.87)	141(8.13)		
Sleep duration per day (hour)			9.827	0.007
< 6	2,211(90.47)	233(9.53)		
6 **∼** 8	19,542(92.21)	1,651(7.79)		
> 8	8,782(91.73)	792(8.27)		
Screen duration per day (hour)			12.940	0.002
< 1	20,304(92.32)	1,689(7.68)		
1 **∼** 3	8,229(91.28)	786(8.72)		
> 3	2,002(90.88)	201(9.12)		
Homework duration per day (hour)			6.913	0.032
< 1	14,569(92.30)	1,216(7.70)		
1 **∼** 3	11,588(91.79)	1,036(8.21)		
> 3	4,378(91.17)	424(8.83)		
Times of breakfast intake per week			12.339	0.006
Never	715(89.26)	86(10.74)		
1 **∼** 2	3,561(91.21)	343(8.79)		
3 **∼** 6	4,584(91.85)	407(8.15)		
Everyday	21,675(92.18)	1,840(7.82)		
Times of meat intake per week			0.828	0.661
< 3	14,313(91.87)	1,267(8.13)		
3 **∼** 4	11,147(92.12)	954(7.88)		
> 4	5,075(91.77)	455(8.23)		
Times of vegetable intake per week			0.278	0.870
< 4	2,603(91.69)	236(8.31)		
4 **∼** 5	7,697(91.95)	674(8.05)		
> 5	20,235(91.97)	1,766(8.03)		
Times of fruit intake per week			1.628	0.443
< 4	7,051(91.71)	637(8.29)		
4 **∼** 5	14,129(91.88)	1,249(8.12)		
> 5	9,355(92.21)	790(7.79)		
Times of eggs intake per week			1.563	0.458
< 4	3,793(91.60)	348(8.40)		
4 **∼** 5	18,624(91.90)	1,642(8.10)		
> 5	8,118(92.21)	686(7.79)		
Times of milk intake per week			0.753	0.686
< 4	4,651(91.66)	423(8.34)		
4 **∼** 5	19,144(91.96)	1,674(8.04)		
> 5	6,740(92.09)	579(7.91)		
Times of fast food consumption per week			7.731	0.021
< 2	20,998(92.22)	1,771(7.78)		
2 **∼** 3	6,947(91.38)	655(8.62)		
> 3	2,590(91.20)	250(8.80)		

### 3.2 Univariable analysis of adolescents physical fitness tests qualified rate

The univariable analysis delineated that several factors were inextricably linked with the physical fitness qualified rate among adolescents aged 12 ∼ 15. These encompassed frequency of passive smoking, whether parents liked physical exercise, whether parents supported children's participation in physical exercise, number of physical exercise sessions per week, duration of each physical exercise session, intensity of physical exercise, sleep duration per day, screen duration per day, homework duration per day, times of breakfast consumption per week, times of fast food consumption per week(P < 0.05) (Table 2).

### 3.3 Multivariable logistic regression analysis of adolescents physical fitness tests qualified rate

Utilizing the insights gleaned from the students' physical fitness tests—defined dichotomously as 0 for qualified and 1 for unqualified—as the dependent variable, and adhering scrupulously to the defined inclusion and exclusion criteria with a significance threshold of P = 0.05, a comprehensive ensemble of 14 independent variables were meticulously incorporated into the multivariable logistic regression analysis. These variables encompassed a spectrum of factors: gender, age, household registration, frequency of passive smoking, whether parents liked physical exercise, whether parents supported children's participation in physical exercise, number of physical exercise sessions per week, duration of each physical exercise session, intensity of physical exercise, sleep duration per day, screen duration per day, homework duration per day, times of breakfast intake per week, times of fast food consumption per week. Subsequent to the analytical rigour imparted by the Hosmer-Lemeshow test, the logistic regression model exhibited a commendable degree of fit (P > 0.05). A deeper delve into the multivariable logistic regression analysis illuminated that, post-adjustment for gender, age, and household registration characteristics, a notably lower odds of failing the physical fitness tests was observed among adolescents whose parents both liked physical exercise (OR = 0.867, 95 % CI: 0.775 ∼ 0.969), whose parents supported children’s participation in physical exercise (OR = 0.839, 95 % CI: 0.726 ∼ 0.970), who participated in physical exercise sessions 3 ∼ 5 times per week (OR = 0.738, 95 % CI: 0.674 ∼ 0.809) or more than 5 times per week (OR = 0.692, 95 % CI: 0.598 ∼ 0.802), who exercised for 0.5 ∼ 1 h each time (OR = 0.768, 95 % CI: 0.701 ∼ 0.842) or more than 1 h each time (OR = 0.754, 95 % CI: 0.650 ∼ 0.875), who engaged in moderate intensity physical exercise (OR = 0.842, 95 % CI: 0.735 ∼ 0.964), who slept 6 ∼ 8 h per day (OR = 0.800, 95 % CI: 0.693 ∼ 0.925) or more than 8 h per day (OR = 0.848, 95 % CI: 0.727 ∼ 0.989), who consumed breakfast 3 ∼ 6 times per week (OR = 0.741, 95 % CI: 0.579 ∼ 0.948) or daily (OR = 0.705, 95 % CI: 0.560 ∼ 0.887). Conversely, an increased odds of failing the physical fitness tests was apparent among adolescents who always exposed to passive smoking (OR = 1.230, 95 % CI: 1.093 **∼** 1.384), who spent 1 ∼ 3 h on screen per day (OR = 1.146, 95 % CI: 1.049 ∼ 1.252) or more than 3 h on screen per day (OR = 1.202, 95 % CI: 1.031 ∼ 1.402), who spent more than 3 h doing homework per day (OR = 1.167, 95 % CI: 1.039 ∼ 1.310), who consumed fast food 2 ∼ 3 times per week (OR = 1.114, 95 % CI: 1.014 ∼ 1.223) or more than 3 times per week (OR = 1.150, 95 % CI: 1.001 ∼ 1.322) (Table 3).

**Table 3:** Multivariable analysis of qualified rate among adolescents aged 12 ∼ 15 in Shandong Province who participated in 2023 physical fitness tests and questionnaire survey.

Independent variables	*OR*(95 %*CI*)	*P*
Gender		
Male	1	
Female	0.917(0.847 **∼** 0.993)	0.033
Age		
12	1	
13	0.951(0.847 **∼** 1.068)	0.397
14	0.944(0.841 **∼** 1.060)	0.329
15	0.853(0.762 **∼** 0.955)	0.006
Household registration		
Urban household registration	1	
Rural household registration	0.918(0.848 **∼** 0.994)	0.035
Frequency of passive smoking		
Never	1	
Sometimes	1.062(0.967 **∼** 1.167)	0.208
Always	1.230(1.093 **∼** 1.384)	0.001
Whether parents liked physical exercise		
Neither side of the parents liked	1	
One side of the parents liked	0.928(0.849 **∼** 1.014)	0.099
Both sides of the parents liked	0.867(0.775 **∼** 0.969)	0.012
Whether parents support children's participation in physical exercise		
Nonsupporting	1	
Supporting	0.839(0.726 **∼** 0.970)	0.018
Number of physical exercise sessions per week (count)		
< 3	1	
3 **∼** 5	0.738(0.674 **∼** 0.809)	0.000
> 5	0.692(0.598 **∼** 0.802)	0.000
Duration of each physical exercise session (hour)		
< 0.5	1	
0.5 **∼** 1	0.768(0.701 **∼** 0.842)	0.000
> 1	0.754(0.650 **∼** 0.875)	0.000
Intensity of physical exercise		
Low intensity	1	
Moderate intensity	0.842(0.735 **∼** 0.964)	0.012
High intensity	0.867(0.699 **∼** 1.075)	0.193
Sleep duration per day (hour)		
< 6	1	
6 **∼** 8	0.800(0.693 **∼** 0.925)	0.003
> 8	0.848(0.727 **∼** 0.989)	0.035
Screen duration per day (hour)		
< 1	1	
1 **∼** 3	1.146(1.049 **∼** 1.252)	0.003
> 3	1.202(1.031 **∼** 1.402)	0.019
Homework duration per day (hour)		
< 1	1	
1 **∼** 3	1.077(0.988 **∼** 1.175)	0.093
> 3	1.167(1.039 **∼** 1.310)	0.009
Times of breakfast intake per week		
Never	1	
1 **∼** 2	0.805(0.626 **∼** 1.034)	0.089
3 **∼** 6	0.741(0.579 **∼** 0.948)	0.017
Everyday	0.705(0.560 **∼** 0.887)	0.003
Times of fast food consumption per week		
< 2	1	
2 **∼** 3	1.114(1.014 **∼** 1.223)	0.025
> 3	1.150(1.001 **∼** 1.322)	0.049

## 4 Discussion

Physical fitness tests scores serve as pivotal markers for gauging the health and developmental progression of adolescents. The government of China, demonstrating an unwavering commitment to the physical well-being of its younger demographic, has consistently given this matter significant attention. As delineated in the “Healthy China 2030″ blueprint, endorsed by the State Council, there is an aspiration to attain an excellence rate exceeding 25 % in national student physical fitness tests by the year 2030 (Dong et al., 2020).

This study delineated the deleterious repercussions of sustained exposure to passive smoking environments on the qualified rate of physical fitness tests of adolescents. Secondhand smoking, colloquially termed “passive smoking,” encompasses the inhalation of tobacco emissions by non-smokers, emanating from individuals engaged in active smoking. A salient observation underscores adolescence as a critical juncture for susceptibility to tobacco smoke (de la Peña et al., 2016). In this demographic subset, prevailing literature intimates a pronounced decrement in respiratory function (Bird and Staines-Orozco, 2016) consequent to secondhand smoke exposure, culminating in attenuated endurance levels. Notably, there is mounting data suggesting potential immune system compromise (Huttunen et al., 2011), augmenting susceptibility to infectious pathogens. Concomitantly, there exists a discernible exacerbation of cardiovascular stress (Wang et al., 2023), predisposing to hypertension (Bucher et al., 2013) and perturbations in heart rate variability (Dinas et al., 2013). A particularly disconcerting finding pertains to the plausible deleterious ramifications of secondhand smoke on adolescents' osteological health (Afghani et al., 2003) and muscular ontogeny (Rom et al., 2013), coupled with potential perturbations in neurodevelopmental trajectories (Freire et al., 2016). The insidious encroachments of passive smoke may extend to holistic health detriments, cognitive impairments, and learning deficits. As such, fortifying this vulnerable demographic against the insalubrious onslaught of secondhand smoke during this vital developmental epoch is imperative.

This investigation revealed a consequential link between parental preferences and endorsement of physical activity, which has a pronounced influence on the qualified rate of physical fitness tests in adolescents. The familial milieu exerts a profound impact on the trajectory of a child's developmental arc. Habitual behaviors, once established during the nascent phases of life, owing to educational and environmental exposures, often solidify, bearing resemblance to inherent traits, and accompany an individual throughout their existence. In a seminal study, the researchers embarked on an exploration of determinants shaping a child's physical exercise tendencies, employing a “lifecycle perspective” (Birchwood et al., 2008). Their insights illuminated that the formative years leading up to the age of 16 wielded an indispensable role in sculpting physical activity dispositions. The ambient conditions and encounters during this pivotal phase emerged as primary catalysts engendering disparities in exercise inclinations. Of particular note was the pronounced emphasis on the family's ambiance, characterizing it as an enduring determinant, resistant to alterations. An augmentation in the family's sports-oriented environment resonates positively with the physical well-being of adolescents (Sánchez-Zamorano et al., 2019). Parental behavioral endorsement emerges as a quintessential gauge, predicting the propensity of adolescents to immerse themselves in physical activities. The perception of parental advocacy for such endeavors often galvanizes children to partake more fervently. Concurrently, a surge in the duration devoted by parents to physical endeavors acts as a catalyst, spurring adolescents to allocate increased time to such pursuits, culminating in enhanced physical health.

This research's elucidation underscored the influence of exercise behaviors - encompassing frequency, duration, and intensity - on the qualified rate of physical fitness tests among adolescents. A robust cognizance coupled with consistent engagement in physical exertions emerges as salutary determinants bolstering the holistic advancement of adolescent physical fitness (Chen et al., 2018). Engaging in physical exercise, adolescents experience multifaceted enhancements in their physiological attributes, spanning endurance, strength, speed, and flexibility. Aerobic modalities, typified by endeavors like running, swimming, and cycling, are evidenced to ameliorate cardiorespiratory functions, thereby augmenting stamina and resilience in middle school pupils (Li and Xu, 2022.). Concurrently, anaerobic regimens such as pull-ups and push-ups amplify muscular vigor and foster salubrious skeletal maturation (Biette et al., 2004). Further, high-velocity anaerobic exercises, akin to sprinting, invigorate the celerity and explosive prowess of adolescents (Markovic et al., 2007). Moreover, flexibility drills, emblematic of gymnastics and yoga, cultivate an expanded joint motility spectrum, attenuate muscular rigidity, and augment physical pliability and agility (Zakas, 2005). Physical exertion's influence on adolescent physical wellness is both profound and invaluable. Recognizing adolescence as a pivotal epoch in psychophysical evolution, physical exercises not only propel growth and maturation trajectories (Hallal et al., 2006) but also hone coordination acumen (Barnett et al., 2016), engender team ethos (Erhardt et al., 2014), and instill a constructive life outlook (Rodriguez-Ayllon et al., 2019). Nonetheless, a salient revelation from this investigation is the marked efficacy of moderate intensity exercises on the enhancement of adolescent fitness. Paradoxically, the amplification to high-intensity regimens failed to manifest congruent positive ramifications. This insinuates that while exercise intensity undeniably modulates adolescent fitness, indiscriminately elevating intensity might not only fall short in achieving desired enhancements in physical fitness but could also precipitate adverse physiological and psychological ramifications, thus impeding optimal adolescent development.

This investigation's outcomes underscored the pivotal influence of sleep duration on the qualified rate in physical fitness tests among adolescents. Sleep, an indispensable physiological phenomenon, assumes paramount importance, especially amidst the adolescent demographic, given their ongoing corporeal growth and maturation. Ensuring sleep sufficiency emerges as vitally crucial (Fonseca et al., 2021). Primarily, during the nocturnal rest phase, there is a marked secretion of growth hormone (Verrillo et al., 2011), instrumental for the adolescent physical trajectory, particularly emphasizing osseous and muscular developments. Additionally, sleep is recognized as a quintessential phase for energy recuperation, crucial for adolescents engrossed in protracted sports and activities. In this dormant phase, the human body orchestrates energy restitution via intricate self-reparative processes (Malhotra, 2017). A deficit in sleep could culminate in pronounced physical lethargy, thereby compromising daily physiological operations.Moreover, sleep is indispensable for the maintenance of neuromuscular coordination and reflexive celerity. A paucity or compromised quality of sleep could result in disrupted neural-muscular synergies, eliciting decelerated reaction paradigms (Louca and Short, 2014). Furthermore, sleep is determinative in memory crystallization and the assimilation of novel competencies (Maquet. 2001). Sleep insufficiency might impinge on the mastery of nascent athletic competencies, such as nuanced technical maneuvers. Lastly, sleep adequacy is of essence for preserving immunological functionalities (Kuna et al., 2022). Optimal sleep fosters a fortified immune architecture in adolescents, underpinning resistance against a plethora of pathologies.

This research elucidated the significant implications of sedentary habits, characterized by extended screen exposure and academic endeavors, on the qualified rate of physical fitness tests within the adolescents. As society evolves, there's an ascendant academic onus on adolescents. Concurrently, the ubiquity of electronic devices has seamlessly amalgamated with quotidian routines, engendering an increasingly sedentary modus vivendi for adolescents, thereby amplifying their duration of inertia. Such sedentary inclinations are now emerging as a burgeoning public health conundrum (Ueno et al., 2022). Primordially, sedentary behaviors are observed to precipitate a diminution in adolescent bone mineral density (Gabel et al., 2017) and instigate muscular atrophy (Gianoudis et al., 2015). Subsequently, protracted periods of seated inactivity, compounded by postural aberrations, may engender orthopedic malalignments in adolescents, typified by scoliosis (Fraiwan et al., 2022). Such deviations not merely mar their aesthetic presentation but could also sow seeds for enduring health complications. Moreover, the inherent nature of a sedentary existence typically denotes attenuated energy expenditures. In scenarios where caloric consumption isn't proportionately moderated, it poses the potential to culminate in weight accretion, thereby exacerbating the susceptibility to overweight and obesity trajectories (Cecchini et al., 2010). Lastly, sedentary postures portend detrimental ramifications on cardiovascular physiology. Chronic adherence to sedentary routines could levy undue strain on the cardiovascular matrix, subsequently escalating the risk indices for chronic maladies, including cardiopathies (Hopkins et al., 2023).

This investigation discerned significant correlations between dietary practices, specifically the regularity of breakfast consumption and fast food intake, and qualified rate of physical fitness tests within the adolescents. Dietary comportment is a salient determinant of an individual's health trajectory (Kärkkäinen et al., 2018), particularly during the formative adolescent phase (Arlinghaus et al., 2020), earmarked by pivotal growth and developmental milestones. The primacy of breakfast in daily dietary regimens is universally acclaimed (Gibney et al., 2018). This inaugural meal serves to recompense for nocturnal energy depletions, recalibrate metabolic processes, and invigorate cognitive faculties such as memory and creativity, with potential reverberations on enhancing exercise efficacy (Vermorel et al., 2003). Given the pronounced metabolic and developmental exigencies of adolescence, consistent bypassing of breakfast could beget nutritional paucities, thereby impinging on their holistic growth trajectory (Mogre et al., 2013). On the other hand, while fast food might be embraced for its expedience, especially by time-pressed adolescents, it typically exhibits nutritional myopia. Predominantly characterized by a surfeit of calories, sugars, salts, and fats, these alimentary choices conspicuously lack the requisite proteins, vitamins, minerals, and fiber, quintessential for balanced nutrition (Khongrangjem et al., 2018). An overreliance on such nutritionally stunted offerings could not only culminate in malnutrition (Beal et al., 2019) but could also precipitate weight augmentations, elevating risks of obesity (Alsabieh et al., 2019).

## 5 Conclusion

Families, educational institutions, and broader society must orchestrate an integrated approach to fortify the physical health trajectory of adolescents, ensuring a harmonious and holistic growth trajectory. Parental engagement, echoing through both active involvement and behavioral role modeling, could be instrumental in instilling robust lifestyle habits in their offspring. Schools, in tandem with familial and societal units, should champion adolescent participation in physical activity, underpinned by provisioning of apt sporting infrastructures and conducive environments. Adolescents should judiciously balance their academic pursuits with relaxation, assuring ample sleep and curtailing protracted sedentary periods. Moreover, a prudent dietary regimen, catering to their evolving nutritional exigencies, warrants emphasis. By entrenching these strategies, we can invigorate the physical constitution of adolescents, buttressing their comprehensive development.

Yet, as with any empirical endeavor, this study unveiled pivotal findings, but it was imperative to discern them in the chiaroscuro of inherent limitations:

(1) Selection bias: Employing stratified random cluster sampling, albeit rigorous, might inadvertently foster representation gaps. The chosen academic establishments might not entirely encapsulate the diversity of the student populace in Shandong Province, underscoring potential discrepancies between included and excluded schools.
(2) Measurement errors: The veracity of self-recounted data, especially gleaned from questionnaires, perennially remains a point of contention. Latent recall biases might skew students' reminiscence of specific endeavors, and the veneer of social acceptability might overshadow their candid revelations.
(3) Cross-sectional design: The cross-sectional design offers but a momentary glimpse, curbing our latitude to extrapolate causative links or chart temporal evolutions.
(4) Elusive confounders: Potentially influential variables—spanning the genetic continuum, environmental factors like ambient air quality, or the ephemeral physical state on the test day—remain beyond the study's purview.
(5) Geographical confinement: Centered on Shandong Province, the study's ambit might not seamlessly transpose to regions or nations ensconced in disparate cultural, socioeconomic, or ecological landscapes.

In synthesizing the study's revelations, it was paramount to weigh these limitations, offering a nuanced lens to understand, interpret, and contextualize the findings.

## 6 Funding statement

This study was supported by a grant from Social Sciences Planning Project of Dongying (DYSK2023No.308).

## CRediT authorship contribution statement

**Zhihao Huang:** Conceptualization, Formal analysis, Methodology, Project administration, Software, Supervision, Writing – original draft, Writing – review & editing. **Shanshan Li:** Conceptualization, Formal analysis, Investigation, Supervision, Visualization, Writing – original draft, Writing – review & editing. **Fei Lu:** Conceptualization, Visualization, Writing – review & editing. **Kunzong Tian:** Methodology, Writing – review & editing. **Lujing Peng:** Formal analysis, Writing – review & editing.

## Declaration of Competing Interest

The authors declare that they have no known competing financial interests or personal relationships that could have appeared to influence the work reported in this paper.

## Data Availability

The data used during the current study are included in this published article and its supplementary information files
