# Supplementary material for: Current situation and factors influencing physical fitness among adolescents aged 12 ∼ 15 in Shandong Province, China: A cross-sectional study
Source: Prev Med Rep. 2023 Oct 12;36:102460. doi: 10.1016/j.pmedr.2023.102460 (PMC10622685; doi:10.1016/j.pmedr.2023.102460)
Supplement: Supplementary data 1 [file mmc1.docx]

**Supplementary Table 1** Questionnaire

questionnaire

Dear participants,

Welcome to our study, which aims to explore the current status of physical fitness and its influencing factors among adolescents aged 12 ~ 15 in Shandong Province. Please take some time to answer the following questions. Please note that this is a completely anonymous questionnaire, and we will not collect any information that can identify your personal identity. There are no right or wrong answers to the questions. We hope you can respond based on your true feelings and actual circumstances.

(1) Gender

⓪ male ① female

(2) Age ___

(3) Household registration

⓪ Urban household registration ① Rural household registration

(4) Family annual income (yuan)

①≤ 100000 ② 100001 **~** 200000 ③ 200001 **~** 300000 ④ ≥ 300000

(5) Paternal education level

① Junior high school or below ② Senior high school ③ Above senior high school

(6) Maternal education level

① Junior high school or below ② Senior high school ③ Above senior high school

(7) Frequency of passive smoking

① Never ② Sometimes ③ Always

(8) Whether parents liked physical exercise

① Neither side of the parents liked ② One side of the parents liked

③ Either side of the parents liked

(9) Whether parents supported children's participation in physical exercise

⓪ Nonsupporting ① Supporting

(10) Number of physical exercise sessions per week

① < 3 ② 3 ~ 5 ③ > 5

(11) Duration of each physical exercise session (hour)

① < 0.5 ② 0.5 ~ 1 ③ > 1

(12) Intensity of physical exercise

① Low intensity ② Moderate intensity ③ High intensity

(13) Sleep duration per day (hour)

① < 6 ② 6 ~ 8 ③ > 8

(14) Screen duration per day (hour)

① < 1 ② 1 ~ 3 ③ > 3

(15) Homework duration per day (hour)

① < 1 ② 1 ~ 3 ③ > 3

(16) Times of breakfast intake per week

① Never ② 1 ~ 2 ③ 3 ~ 6 ④ Everyday

(17) Times of meat intake per week

① < 3 ② 3 ~ 4 ③ > 4

(18) Times of vegetable intake per week

① < 4 ② 4 ~ 5 ③ > 5

(19) Times of fruit intake per week

① < 4 ② 4 ~ 5 ③ > 5

(20) Times of eggs intake per week

① < 4 ② 4 ~ 5 ③ > 5

(21) Times of milk intake per week

① < 4 ② 4 ~ 5 ③ > 5

(22) Times of fast food consumption per week

① < 2 ② 2 ~ 3 ③ > 3
